# Scientific Items

**Published:** 1877-05-20

**Authors:** 


### Scientific Items.

MALLEABILITY OF GOLD.–A single grian of gold may be beaten out so as to cover a space of seventy-five square inches, the thickness of the leaf is then only the three hundred and sixty-seven thousand six hundred and fiftieth part of an inch.

THE PLANET JUPITER.—Recent observations indicate that the real body, or nucleus of the planet Jupiter has never been seen, it being surrounded by a semi-transparent atmosphere of about 11,000 miles in depth, in which float vapors and vast cloud masses, sometimes in layers, at others in irregular heaps, having well rounded forms. It is believed that deep down below these vast cloudy layers lies the fiery mass of the real planet, in which are upheavals and outbursts compared with which the most tremendous volcanic explosions on our earth are utterly insignificant.

EVOLUTION.—Sir Wyville Thompson said before the natural history class at Edinburgh: "We have in fossil remains a record of the inhabitants of this world running back incalculably farther than man's existence on this planet, and although we find that thousands of species have passed away, and thousands have appeared, in no single case have we yet found the series of transitional forms imperceptibly gliding into one another, and uniting two clearly distinct species by a continuous bridge, which could be cited as an undoubted instance of the origin of a species."

THE quantity of air inhaled by an adult in 24 hours averages about 360 cubic feet, or 2,000 gallons—about 730-000 gallons in one year.

